# Ethnotherapeutic Uses and Phytochemical Composition of *Physalis peruviana* L.: An Overview

**DOI:** 10.1155/2021/5212348

**Published:** 2021-10-11

**Authors:** Félicien Mushagalusa Kasali, Jonans Tusiimire, Justin Ntokamunda Kadima, Casim Umba Tolo, Anke Weisheit, Amon Ganafa Agaba

**Affiliations:** ^1^Pharm-Bio Technology and Traditional Medicine Center (PHARMBIOTRAC), Mbarara University of Science and Technology, P.O. Box 1410, Mbarara, Uganda; ^2^Department of Pharmacy, Faculty of Pharmaceutical Sciences and Public Health, Official University of Bukavu, P.O. Box 570, Bukavu, Congo; ^3^Department of Pharmacy, Faculty of Medicine, Mbarara University of Science and Technology, P.O. Box 1410, Mbarara, Uganda; ^4^Department of Pharmacology, School of Medicine and Pharmacy, University of Rwanda, P.O. Box 117 Huye, Kigali, Rwanda; ^5^Department of Pharmacology and Therapeutics, Faculty of Medicine, Mbarara University of Science and Technology, P.O. Box 1410, Mbarara, Uganda

## Abstract

**Background:**

Plant-derived medicines are widespread and continue to increase in traditional and modern medicine, especially in developing countries. *Physalis peruviana* L. is among the most used plants in conventional medication worldwide. This review aimed to highlight the ethnotherapeutic uses and phytochemical status of identified compounds in *P*. *peruviana*.

**Methods:**

Data were collected from Google Scholar, PubMed/Medline, SciFinder, Science Direct, Scopus, the Wiley Online Library, Web of Science, and any other helpful search engine using *Physalis peruviana* as the primary keyword.

**Results:**

Some countries, worldwide, use *P*. *peruviana* in their traditional medicine system to manage diverse ailments, mainly diseases and gastrointestinal tract disorders (25.33%). Leaf was the mostly used part (49.28%), prepared by decoction (31.58%) and overall administrated orally (53.57%) as the main route of admission. Around 502 phytoconstituents were identified in different plant parts, especially fruit (38.19%) ethanol/ethyl acetate extract. In most cases (36.17%), the solvent of the extract was not specified. Several phytochemical classes were found in the plant, especially terpenes (26.09%) and phenolic compounds (14.94%). Esters were also abundant (11.55%). In the terpenes category, carotenoids were the most abundant (11.15% followed by monoterpenes (8.76%) and diterpenes (3.18%). However, flavonoids (5.17%) followed by cinnamic acid derivatives (3.99%), monophenolic compounds (1.79%), and phenolic acids (1.33 M) are the most reported phenolic compounds. Hexadecanoic acid (palmitic acid) was the most cited (five times).

**Conclusion:**

*P*. *peruviana* plays an essential role in managing diseases in some countries and is rich in chemical compounds, which need to be isolated and investigated pharmacologically before clinical trials.

## 1. Introduction

According to the World Health Organization (WHO), about 80% of the population in developing countries uses herbal medicine to meet their primary healthcare requirements [1]. Humans have used natural products since prehistoric times, which include animals, marine organisms, microorganisms, and plants, in medicines to prevent, diagnose, and treat diseases [2]. Plants still contribute primarily to health care, so many specific herbal extracts have been demonstrated to be productive for particular conditions [3]. More than 50,000 plants would possess therapeutic virtues globally. In Africa and Asia, it is estimated that more than 80 percent of the population uses traditional medicine for primary health care. This form of therapy remains prevalent in all world regions, and its use is rapidly spreading in developed countries [4].

*Physalis peruviana* (Solanaceae) is a native plant from the Andean region and a semiupright herbaceous shrub or perennial, producing a group of branched stems native to the Andean region. *P*. *peruviana* is adapted to a wide range of altitudes, soils, and climatic conditions. It is also the most widely distributed species from the *Physalis*. *Physalis* genus contains several species with a long history of ethnomedical use to treat diverse diseases, especially asthma, cancer, dermatitis, hepatitis, bacterial infections, kidney and liver disorders, and malaria and has immunomodulatory antipyretic properties [5]. It contains different types of compounds, including physalins and alkaloids, flavonoids, carotenoids, vitamins, and polysaccharides [6, 7]. The health benefits of the plant are related to the content of phytochemicals.

This report summarizes ethnomedicinal use and phytoconstituents identified in *P*. *peruviana*. Previous reviews have been focused on nutritional values, pharmacological evidence, and phytochemical profiling of isolated compounds from the plant [8].

This review aimed to highlight the ethnotherapeutic use and phytochemical status of identified compounds in *P*. *peruviana L*.

## 2. Literature Review Method

Different search databases, including Google Scholar, PubMed/Medline, Science Direct, Scopus, the Wiley Online Library, Web of Science, and any other helpful search engines using *P*. *peruviana* as the primary keyword, were used. Full articles in English or French languages were retrieved without time limit restriction.

## 3. Results and Discussion

### 3.1. Ethnopharmacological Data of *P. peruviana* L

The following Table 1 presents the uses of *P*. *peruviana* in traditional medicines in different countries.

According to this table (Table 1), fourteen countries worldwide use *P*. *peruviana* in their traditional medicinal system to treat several diseases. India represented the most cited country with twelve references, followed by Uganda (10), Kenya (7), Cameroon, Democratic Republic of Congo, Nepal, South Africa, and Tanzania, each with three references. Colombia and Indonesia were cited only twice.

Referring to the number of diseases treated by country, India is the most representative country (20.27%), followed by Uganda (16.22%), the Democratic Republic of Congo (12.16%), Cameroon (6.76%), Colombia, Nepal, and South Africa (5.41%). It is known that the plant (mainly le fruit) is produced predominantly in Colombia and South Africa but exported in Netherlands, Germany, Belgium, and Canada [61]. However, its use in traditional medicine is widespread in other countries, including India and Uganda.

The plant is widely known in various local names and used in Ayurvedic medicine for many human and animal purposes. The fruit is available from January to April [62]. In Uganda, the plant grows naturally in abandoned bush fallows, and it is helpful for income. It has been identified as a priority plant for commercialization (used popularly for its berries and associated derivative products such as juice, jam, and wine). It is also used as food and has medicinal applications [63].

Local names, parts used, traditional utilization, preparation, and administration modes were documented.

**Figure 1:**
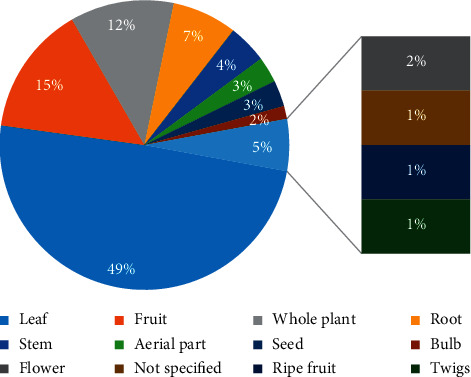
Frequencies of parts used.

Figure 1 indicates that the leaves are the most used part (49.28%) followed by fruits (14.49%), whole plant (11.59%), roots (7.5%), stem (4.35%), aerial parts, and seeds (2.90%). However, bulbs, flowers, ripe fruits, and twigs were cited once (1.45%). In some cases, the used parts were not specified (1.45%). Leaves are the most used in the formulation of remedies, as indicated above. The frequent use of leaves is associated with ease of accessibility among the aboveground parts of plants in natural ecosystems [50].

**Figure 2:**
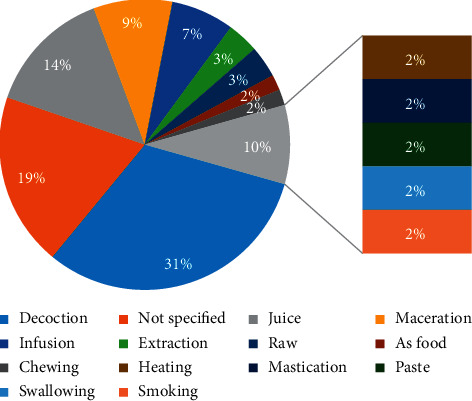
Frequencies of formulations.

Decoction has often been found as the effective formulation of herbal remedies as it is easy to prepare by mixing a drug with boiling water [64]. In this study (Figure 2), the decoction was used in almost 31.58% of all cases. However, other preparation modes have been found including juice (14.04%), maceration (8.77%), infusion (7.02%), extraction, and raw material (3.51%). In 19.30% of cases, the preparation mode was not reported.

**Figure 3:**
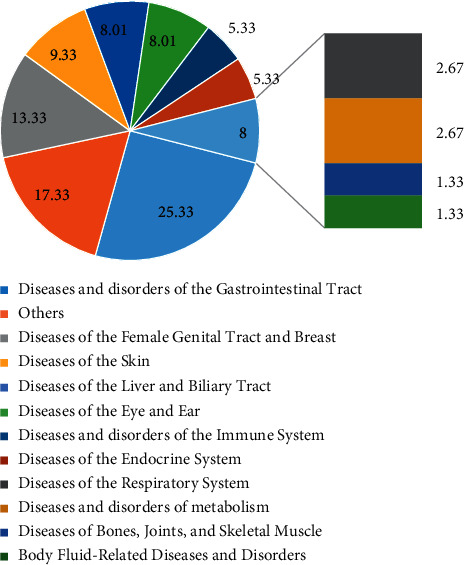
Frequencies of diseases and disorders treated.

*P*. *peruviana* is indicated to treat various diseases, mainly in humans. Rarely, it is used in the management of diseases in veterinary medicine. For example, in western Kenya, it is used for livestock tick prevention and control. The results in Figure 3 show that diseases and disorders of the gastrointestinal tract were the most treated by the plant (25.33%), followed by female genital tract and breast (13.33%), skin (9.33%), liver and biliary tract (8.01%), eye and ear (8.01%), immune system (5.33%), endocrine system (5.33%), respiratory system (2.67%), and metabolic disorders (2.67%). Diseases of bones, joints, skeletal muscle, and body fluid-related diseases and disorders represent 1.33%. Another category of diseases, including helminthiasis, inflammations, malaria, snake bite, fungal infections, bacterial infections, and smallpox, represents 17.33%. About 4000 species had ethnomedical data supporting the use of these plants to treat, and most of them were native to tropical countries due to the extraordinary biodiversity in these countries [65].

**Figure 4:**
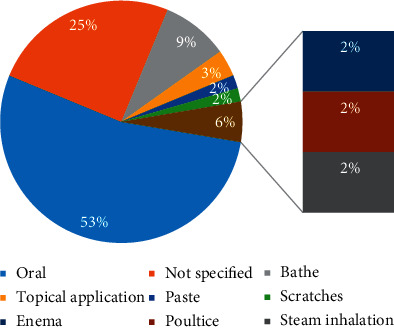
Frequencies of routes of administration.

Mostly, oral route is the way of drug administration based on different formulations. Because of safety, good patient compliance, ease of ingestion, pain avoidance, and versatility to accommodate various types of drugs, the oral administration route is preferred over the different other administration routes of drug delivery [66]. Nevertheless, the route of the administration is not specified in a few cases (20.41%). Secondarily, bathe, tropical application, scratches, and steam inhalation are reported (Figure 4).

There are some specific indications in formulations or modes of drug administration. For example, in India, the plant is associated with *Impatiens roylei* and *Stephania hernandifolia* to treat jaundice. In the same way, in Uganda, the plant is combined with *Solanum esculentum* and *Solanum melongena* to manage skin problems in babies and honey in treating malaria. It is possible that combining several plants can produce a more pronounced pharmacological response than using a single plant due to the synergy of action between different constituents. According to Sofowora et al. [67], the combined effects were much more effective than individual ones. Rarely, duration of treatment and posology were mentioned. However, those two factors depended on the type of diseases treated and the parts used. For example, in Uganda, treating malaria needs seven days by taking two teaspoons three times a day of a decoction or half a glass thrice a day. In Tanzania, an application of leaf juice on the affected area twice a day was indicated to treat skin fungal infections or heating/topical application on to cuts and scratches in New Guinea for boils and ulcers. In Nepal, the treatment of jaundice in children could take from four to ten days.

The voucher number of plant material was not specified in 63.46% against 36.54%. Overall, in research studies that involved plant or animal materials, providing voucher specimens is necessary for several reasons. The main reason is to keep a permanent record documenting the plant used in a specific study to trace the true identity and source of the plant material [68]. In most cases, the plant species look alike (morphologically and chemically), and it is quite possible to have a confusing error when harvesting. To be reassured of the real identity of the plant, it is crucial to have it authenticated with an expert, for example, a botanist. In the event of a future contestation, the voucher number recorded in the herbarium will always be essential to confirm the integrity of its identity. It is also vital for reproducibility, which is very critical in research.

### 3.2. Phytoconstituents Identified in Different Parts of P. peruviana L

Table 2 summarizes the chemical compounds identified and characterized from other parts and extracts of *P*. *peruviana*. Therefore, various classes of phytoconstituents have been found, including terpenes (monoterpenes, sesquiterpenes, diterpenes, triterpenes, and carotenoids), phenolic compounds (phenolic acids, phenolic esters, phenolic aldehydes, chalcones, coumarins, cinnamic acid derivatives, flavonoids, and glucosides), alcohols, aldehydes, ketones, carboxylic acids, lactones, steroids and withanolides, alkaloids, sucrose esters, glucosides, siloxanes, vitamins, phytoprostanes, phytol derivatives, enols, heterocycles, alkanes, alkenes, benzimidazoles, and diverse functional groups.

Different parts of *P*. *peruviana* contain terpenes, and polyphenols represent the main two classes of identified phytoconstituents. They represent 26.09% and 14.94%, respectively. In the terpenes category, carotenoids are the most representative (11.15%), followed by monoterpenes (8.76%), sesquiterpenes (5.57%), and diterpenes (3.18%). A considerable amount of sesquiterpenes (22.3%) and fatty acids (22.8%) has been found in *P*. *angulata*, a *Physalis* species close to *P*. *peruviana*, as volatile components of leaf essential oil [91]. However, phytol (17.88%) was the most diterpenes found in ethanolic extracts of leaves, roots, and fruits of *P*. *minima*, beyond other phytoconstituents, including fatty acids [92]. According to our results, phytol was identified right now, only in calyces and leaves of *P*. *peruviana*.

The presence of phytoene can justify the richness of the plant in carotenoids. Therefore, phytoene is an alkene hydrocarbon with 40 carbon atoms intermediate in the biosynthesis of carotenoids. The synthesis of phytoene is necessary for that of carotenoids in plants. The biosynthetic pathway from phytoene to violaxanthin is common to the genus *Physalis* [70]. Furthermore, carotenoid pigments from different species of the *Physalis* genus are primarily used in the food industry as food dyes for fats and oils. Their seeds can contain up to 30% fatty oil [93]. The presence of carotenoids in the *Physalis* genus has been confirmed by Ramadan [94]. All-*trans*-*β*-carotene, 9-*cis*-*β*-carotene, and all-*trans*-*α*-cryptoxanthin were the primary carotenoids found in the fruits.

Referring to phenolic compounds, flavonoids are the most phytoconstituents found (5.17%) in the plant than cinnamic acid derivatives (3.98%), monophenolic compounds (1.79%), phenolic acids (1.39%), coumarins (0.79%), phenolic esters (0.79%), chalcones (0.39%), phenolic aldehydes (0.39%), and stilbenes (0.19%). Similarly, phenolic, flavonoid, and phenolic acid contents were identified and quantified in different parts of five members of the *Physalis* genus including *P*. *angulate*, *P*. *patula*, *P*. *subulata*, *P*. *solanacea*, and *P*. *hederifolia*. However, quercetin, kaempferol, and phenolic acids were identified as the major phenolic phytoconstituents in those five plant species, in different concentrations according to organs [95]. Overall, monophenolic and polyphenolic compounds are synthesized and then accumulated in all plant tissues, but their concentration can be varied from different parts. Among phenolic compounds, phenolic acids and flavonoids are the most studied, mainly pharmacological properties exploited for medical purposes [96]. Gupta et al. [97] noted the strong influence of phenolic compounds and the carotenoid content with bioactivity.

The plant also contains fatty acids, which are the most cited in the literature. For example, hexadecanoic acid (palmitic acid) was the most cited, five times (0.82%), followed by decanoic acid, linoleic acid, and octadecanoic acid, which were mentioned four times (0.66%). Hexadecanoic acid (palmitic acid) is the most common saturated fatty acid in plants, animals, and microorganisms, and linoleic acid is central in plant lipids. It is essential for humans (animals) because it is derived mainly from dietary plant oils [98].

Beyond the sucrose esters identified in plants (2.58%), others such as peruvioses A, B, C, D, and F had already been isolated before in the dichloromethane extract of the sticky exudate that covers the fruit [99, 100]. Nicandroses, other sucrose esters, have been isolated in the *Physalis* genus. Their presence is confirmed in different species including *P*. *nicandroides* var. *attenuata*, *P*. *solanaceus*, *P*. *sordida*, and *P*. *viscosa* [5].

Steroids and withanolides (a group of naturally occurring polyoxygenated steroidal lactones) were also identified in the plant and represented 6.97%. Physalins (steroidal constituents) are the most active representatives of secondary metabolites of the genus [101]. Most withanolide compounds are produced by Solanaceae plants, in particular 19 genera of Solanaceae, including *Acnistus*, *Datura*, *Deprea*, *Dunalis*, *Discopodium*, *Exodeconus*, *Hyoscyamus*, *Iochroma*, *Jaborosa*, *Larnax*, *Lycium*, *Nicandra*, *Physalis*, *Salpichroa*, *Trechonaetes*, *Tubocapsicum*, *Vassobia*, *Withania*, and *Witheringia* [102, 103]. Nowadays, several withanolides have been isolated and characterized from different parts of *P*. *peruviana*, including dihydrowithaferins, physachenolides, physacoztolides, perulactones, withaperuvins, alkekenginins, withaferins, hydroxy-withanolides, physagulins, withaperuvins, physalolactones, withalongolide, physapubescins, withaphysanolides, viscosalactones, and phyperunolides [5, 8]. Almost 351 withanolides have been identified and isolated from the *Physalis* genus, mainly from *P*. *peruviana* and *P*. *angulata* [104].

Steroids such as ergosterol, campesterol, stigmasterol, lanosterol, *ß*-sitosterol, Δ5-avenasterol, and Δ7-avenasterol have been reported in *P*. *peruviana* pomace and fruit juice. A number of the vitamins have been identified primarily in pomace and fruits, including 1,25-dihydroxy vitamin D2 (derived from vitamin D), vitamin B9 (folic acid), vitamin K, vitamin E (*α,β,γ,δ*-tocopherols), and biotin. A study on the phytochemical composition of goldenberry pomace confirmed the presence of those vitamins. In addition to vitamins A, D, and K, niacin, riboflavin, thiamin, pyridoxine, vitamin B12, choline chloride, and *p*-aminobenzoic acid have been identified and quantified [105, 106].

Among ten alkaloids identified in the plant, cuscohygrine was subsequently isolated from the roots [107], and physoperuvine has already been isolated from *P*. *peruviana* roots [108]. The other alkaloids have been explicitly isolated in the aerial and roots. They are the only parts of plants where alkaloids were identified.

## 4. Conclusion

*P*. *peruviana* plays a significant role in managing various pathologies of different organ systems, but its ethnotherapeutic use is strongly limited to a few countries. The plant is very rich in compounds, considering the number of identified compounds. Regarding phytochemical profiling, effort must be directed towards isolating and characterizing more compounds, particularly those that can present a significant therapeutic interest via extensive pharmacological investigations.

## 5. Disclosure

This study is part of the Ph.D. training of FMK. The funding agent had no role in the study design, data collection, data analysis, and writing of the present manuscript.

## Figures and Tables

**Table 1:** Ethnomedicinal uses of *P*. *peruviana* L. in different countries.

Countries	Vernacular names	Part(s) used	Traditional uses	Formulation/method of administration	Voucher number	References
Cameroon	—	Twigs	Cancer or disease relevance to cancer or cancer-like symptoms	—	Yes	[9]
	Ajijieuh	Leaf and stem	Bile, swelling of legs and ankles for pregnant women	Maceration/oral	Yes	[10]
	Ma pe pie	Leaf and stem	Fungal infections	Maceration/oral	Yes	[11]

Colombia	Uchuva	Fruit	Ear pain and diabetes	—	No	[12]
	Uchuva	Fruit	Conjunctivitis and prevention of cataract	Juice/oral	No	[13]

Equator	Uvilla	Flower	Disinfectant and healing of wounds	Decoction/bathe	Yes	[14]

Democratic Republic of the Congo	Mbuma	Leaf	Malaria, intestinal worms, and splenomegaly	Decoction and infusion/oral	Yes	[15]
	Mbuma, Mbupuru, Umuhire	Aerial part	Diabetes mellitus, colic in children, spleen, malaria, and			

inflammation	Decoction/oral	No	[16]			
	Mpuhuhu	Whole plant	Helminthiasis	Maceration/oral	Yes	[17]
India	Donam as	Fruit	Gastric	Mastication/oral	No	[18]
	Fatki	Leaf and root	Leucorrhea and hydrocele	Decoction/oral	No	[19]
	Kitutu	Leaf	Induction of labor and ease childbirth	Decoction/oral	No	[20]
	Kopalphoota	Whole plant	Jaundice	Raw/oral	No	[21]
	Phakphake	Ripe fruit	Throat sore	Mastication/oral	Yes 0032	[22]
	Pottipalam	Leaf and dried seed	Jaundice and glaucoma	—	No	[23]
	Rashbhari	Leaf	Abdominal disorder during pregnancy	Juice/oral	No	[24]
	Tsiibobopro	Leaf and fruit	Diarrhea and dysentery	Decoction and raw/oral	No	[25]
	Sodukku thakkali	Whole plant	Skin diseases	Extraction/-	No	[26]
	—	Leave	Jaundice	Decoction/oral	No	[27]
	—	Whole plant	Gout	—	No	[28]
	—	Leaf	Jaundice	Paste/-	Yes	[29]

Indonesia	Depuk-depuk	Fruit and whole plant	Smallpox	Decoction/oral	No	[30]
	Pultak-pulta	All parts of the plant	Stomach ache	Decoction/oral	No	[31]

Java	Ciplukan	Leaf and fruit	Diabetes mellitus	—	No	[32]

Kenya	Embunwe, emiilwa (wanga)	Stem, root, fruit, and leaf	Inflammation and abdominal ailments^Ψ^	Raw and infusion/poultices and enema	No	[33]
	Mayengo	Leaf	Malaria	Decoction/steam inhalation	Yes	[34]
	Mŭnathi	Leaf	Postpartum pain	Decoction/oral	No	[35]
	Mŭnathi	Leaf	Anthelmintic, postpartum pains, and typhoid	—	No	[36]
	Mŭnathi	Seed, bulb, fruit, leaf, and root	Diarrhea	—	No	[37]
	—	Leaf	Diabetes, malaria, and pneumonia	Decoction/oral	No	[38]
	—	Leaf	Typhoid and pneumonia	Decoction/-	No	[39]

Nepal	Gangathopa	Root	Jaundice	Maceration/oral	No	[40]
	Ram bhutka, Jangali mewa	Root	Piles	—	No	[41]
	—	Leaf	Sore throat and abdominal pain	Juice/oral	No	[42]

New Guinea	Mondon	Leaf	Boils and ulcers	Heating/topical application on to cuts and scratches^*∗*^	No	[43]

Rwanda	Umuhuhu	Leaf	Facilitates the issuance of the placenta and abortifacient	—	No	[44]

South Africa	Igquzu	Leaf	Diarrhea and associated ailments.	As food/oral	No	[45]
	Igquzu	Leaf	Diarrhea	Decoction/oral	Yes	[46]
	—	Whole plant and leaf	Cancer	Decoction/oral	DS00095	[47]
Tanzania	Kitutun kikubwa	Leaf	Malaria	Maceration/oral	Yes	[48]
	Msupu	Leaf	Skin fungal infections	Juice/topical application^*∗*^	Yes	[49]
	Ntuntunu	Fruit	Typhoid fever	Juice/oral	Yes	[50]

Uganda	Entuutu	Leaf and fruit	Snakebite	Infusion/oral	No	[51]
	Entuutu	Fresh leaf	Skin problems in babies	Decoction/bathe^*∗*^	No	[52]
	Entuutu	—	Wounds (fresh)	—	No	[53]
	Kitutu	Leaf	Induce of labor during childbirth	Juice/oral	No	[54]
	Ntuntunu enene	Leaf	Vomiting	Smoked and infusion/bathe	Yes	[55]
	Ntuntunu	Leaf	Malaria	Decoction/oral^*∗*^	Yes	[56]
				Decoction/oral^*∗*^		
	Ntuntunu	Leaf	Infections (antibacterial)	Juice/oral	No	[57]
	Ntutunu enene	Fruit	Ear and eye infection	Chewing and swallowing/oral	Yes	[58]
	Ntutunu enene	Aerial part and leaf	HIV/AIDS	—	Yes	[59]
	—	Whole plant	Rash and ringworm	Juice/-	Yes	[60]

Veterinary use (Ψ); specific characteristics (^*∗*^); not specified (—).

**Table 2:** Chemical compounds identified from different parts and extracts of *P*. *peruviana*.

Organs	Phytoconstituents	Source	References
*Aerial parts*	3*α*-Tigloylnxytropane	Ethanol	[69]
	3*β*-Acetoxytropane	Ethanol	[69]
	Antheraxanthin	Hexane/acetone/ethanol	[70]
	Cuscohygrine	Ethanol	[69]
	Hygrine	Ethanol	[69]
	Lutein	Hexane/acetone/ethanol	[70]
	Neoxanthin	Hexane/acetone/ethanol	[70]
	N-Methylpyrrolidinylhygrine A	Ethanol	[69]
	N-Methylpyrrolidinylhygrine B	Ethanol	[69]
	Physoperuvine	Ethanol	[69]
	Phytofluene	Hexane/acetone/ethanol	[70]
	Tropine	Ethanol	[69]
	Violaxanthin	Hexane/acetone/ethanol	[70]
	Zeaxanthin	Hexane/acetone/ethanol	[70]
	*γ*-Carotene	Hexane/acetone/ethanol	[70]

*Body*	(S)-4-Iodo-1,2-epoxybutane	—	[71]
	1,1,1,5,7,7,7-Heptamethyl-3,3 bis(trimethylsiloxy) tetrasiloxane	—	[71]
	1,2,3-Tri(t-butyl) cyclopropenylium tribromide	—	[71]
	1,2-Benzenedicarboxylic acid	—	[71]
	3,3-Dimethyl-hexane	—	[71]
	3,3-Dimethyl-octane	—	[71]
	Diethyl ester	—	[71]
	Docosane	—	[71]
	Eicosamethyl cyclodecasiloxane	—	[71]
	Eicosamethyl cyclodecasiloxane	—	[71]

*Calyces*	(all-*E*)-Lutein	Hexane/acetone/ethanol	[72]
	(all-*E*)-Lutein 3-O-myristate	Hexane/acetone/ethanol	[72]
	(all-*E*)-Neoxanthin	Hexane/acetone/ethanol	[72]
	(all-*E*)-Neoxanthin palmitate	Hexane/acetone/ethanol	[72]
	(all-*E*)-Taraxanthin	Hexane/acetone/ethanol	[72]
	(all-*E*)-Taraxanthin ester	Hexane/acetone/ethanol	[72]
	(all-*E*)-Violaxanthin	Hexane/acetone/ethanol	[72]
	(all-*E*)-Violaxanthin or (all-*E*)-neoxanthin ester	Hexane/acetone/ethanol	[72]
	(all-*E*)-*α*-Carotene	Hexane/acetone/ethanol	[72]
	(all-*E*)-*α*-Cryptoxanthin myristate	Hexane/acetone/ethanol	[72]
	(*E*)-Vanillic acid	Ethyl acetate	[73]
	(*E*)-*α*-Carotene	Hexane/acetone/ethanol	[72]
	(*Z*)-Lutein 1	Hexane/acetone/ethanol	[72]
	(*Z*)-Lutein 2	Hexane/acetone/ethanol	[72]
	(*Z*)-Lutein ester	Hexane/acetone/ethanol	[72]
	(*Z*)-Taraxanthin	Hexane/acetone/ethanol	[72]
	(*Z*)-Taraxanthin-*⍺*-linolenic acid	Hexane/acetone/ethanol	[72]
	(*Z*)-*β*-Carotene	Hexane/acetone/ethanol	[72]
	*⍺*-Copaeneol	Ethanol/ethyl acetate	[74]
	13-Epimanool	Ethanol/ethyl acetate	[74]
	16-B_1_-PhytoP	Ethanol/ethyl acetate	[74]
	16*α*-Methylpregnenolone	Ethanol/ethyl acetate	[74]
	17,27-Dihydroxylated withaloid D isomer 1	Ethanol/ethyl acetate	[74]
	2,3-Dihydro-17,27-hydroxylated withanolide D derivative	Ethanol/ethyl acetate	[74]
	2,3-Dihydro-27-hydroxylated withanolide D isomer 1	Ethanol/ethyl acetate	[74]
	2,3-Dihydro-27-hydroxylated withanolide D isomer 2	Ethanol/ethyl acetate	[74]
	2,3-Dihydro-27-hydroxy-4*β*- hydroxywithanolide E isomer	Ethanol/ethyl acetate	[74]
	2,3-Dihydro-4*β*-hydroxywithanolide E	Ethanol/ethyl acetate	[74]
	2,3-Dihydro-hydroxylated 4*β*-hydroxywithanolide E derivative	Ethanol/ethyl acetate	[74]
	2′,4′-Dimethoxy-3-hydroxy-6-methylflavone	Methanol	[75]
	2′,5′-Dimethoxyflavone	Methanol	[75]
	27-Hydroxy-4*β*-hydroxywithanolide E isomer	Ethanol/ethyl acetate	[74]
	2-Hydroxy-2′,4′,6′-trimethoxychalcone	Methanol	[75]
	3-(3,4-Dimethoxyphenyl)-6-methyl-4-phenylcoumarin	Methanol	[75]
	3-(3,4-Dimethoxyphenyl)-7- hydroxy-4-methylcoumarin	Methanol	[75]
	3,2′,4′,5′,6-Pentamethoxyflavone	Methanol	[75]
	3,4,5-Methoxy cinnamic	Ethyl acetate	[73]
	3,5,3′,5′-Tetra-*tert*-butyldiphenoquinone	Methanol	[75]
	3,6,2′,3′-Tetramethoxyflavone	Methanol	[75]
	3,6,3′,4′-Tetramethoxyflavone	Methanol	[75]
	3′-Benzyloxy-5,6,7,4′-tetramethoxyflavone	Methanol	[75]
	3-Hydroxy-7,8,2′-trimethoxyflavone	Methanol	[75]
	3-O-Caffeoylquinic acid	Methanol/water/formic acid	[76]
	3-O-Feruloylquinic acid	Methanol/water/formic acid	[76]
	3-O-*p*-Coumaroylquinic acid	Methanol/water/formic acid	[76]
	4,4-Dimethyl-5-*α*-cholestane-3-one	Ethanol/ethyl acetate	[74]
	4-Aminobenzoic acid	Ethyl acetate	[73]
	4-Hydroxy chalcone	Methanol	[75]
	4-O-Feruloylquinic acid	Methanol/water/formic acid	[76]
	5-(7a-Isopropenyl-4,5-dimethyl-octahydroinden-4-yl)- 3-methyl-pent-2-en-1-ol	Ethanol/ethyl acetate	[74]
	5,6-Epoxy-*β*-carotene	Hexane/acetone/ethanol	[72]
	5-O-Caffeoylquinic acid (chlorogenic acid)	Methanol/water/formic acid	[76]
	5-O-Feruloylquinic acid	Methanol/water/formic acid	[76]
	7-Hydroxycoumarin-3- carboxylic acid	Methanol	[75]
	7*δ*-Ergosterol	Ethanol/Ethyl acetate	[74]
	9-D_1t_-PhytoP	Methanol	[76]
	9-Epi-9-D_1t_-PhytoP	Methanol	[76]
	9-Epi-9-F_1t_-PhytoP	Methanol	[76]
	9-F_1t_-PhytoP	Methanol	[76]
	9-L_1_-PhytoP	Methanol	[76]
	Acecetin	Ethyl acetate	[73]
	Ambrial	Ethanol/ethyl acetate	[74]
	Apg 6 arabinose 8 glucose	Ethyl acetate	[73]
	Apg 6 glucose 8 rhamnose	Ethyl acetate	[73]
	Apg 6 rhamnose 8 glucose	Ethyl acetate	[73]
	Apig-7-O-neohespiroside	Ethyl acetate	[73]
	Apigenin	Ethyl acetate	[73]
	Apigenin 7 glucose	Ethyl acetate	[73]
	Benzoic acid	Ethanol/Ethyl acetate	[74]
	Biotin	Methanol	[75]
	Caffeic acid	Ethanol/Ethyl acetate	[74]
	Caffeine	Ethyl acetate	[73]
	Catechol	Ethyl acetate	[73]
	Chlorogenic acid	Ethyl acetate	[73]
	Chlorophyll a	Hexane/acetone/ethanol	[72]
	Chlorophyll a derivative	Hexane/acetone/ethanol	[72]
	Chlorophyll b	Hexane/acetone/ethanol	[72]
	Chlorophyll b derivative 2	Hexane/acetone/ethanol	[72]
	Cinnamic acid	Ethyl acetate	[73]
	Coniferol	Ethanol/ethyl acetate	[74]
	Copalol isomer 1	Ethanol/ethyl acetate	[74]
	Copalol isomer 2	Ethanol/ethyl acetate	[74]
	Copalol isomer 3	Ethanol/ethyl acetate	[74]
	Coumarin	Ethyl acetate	[73]
	Cryptomeridiol	Ethanol/ethyl acetate	[74]
	Diepicedrene-1-oxide	Ethanol/ethyl acetate	[74]
	Dihydro-4*β*-hydroxywithanolide E	Ethanol/ethyl acetate	[74]
	Dihydromanoyl oxide 1	Ethanol/ethyl acetate	[74]
	Dihydromanoyl oxide 2	Ethanol/ethyl acetate	[74]
	Dihydromanoyl oxide 3	Ethanol/ethyl acetate	[74]
	Dihydromanoyl oxide 4	Ethanol/ethyl acetate	[74]
	Dihydromanoyloxide-7-carboxylic acid methyl ester	Ethanol/ethyl acetate	[74]
	Di-O-isobutanoyl-O-(2-methylbutanoyl)-O-pentenoylsucrose	Ethanol/ethyl acetate	[74]
	Di-O-isobutanoylsucrose	Ethanol/ethyl acetate	[74]
	Di-O-isobutanoyl-O-nonanoylsucrose	Ethanol/ethyl acetate	[74]
	Di-O-isobutanoyl-O-decanoylsucrose	Ethanol/ethyl acetate	[74]
	Di-O-isobutanoyl-O-octanoylsucrose	Ethanol/ethyl acetate	[74]
	Di-O-isobutanoyl-O-pentenoylsucrose	Ethanol/ethyl acetate	[74]
	Ellagic acid	Ethyl acetate	[73]
	Ent-16-B1-PhytoP	Methanol	[76]
	Ent-9-L1-PhytoP	Methanol	[76]
	Ent-16-epi-16-F1t-PhytoP	Methanol	[76]
	Ent-16-F1t-PhytoP	Methanol	[76]
	Epicatechin	Ethyl acetate	[73]
	Epimanoyl oxide	Ethanol/ethyl acetate	[74]
	Eudesmadienol	Ethanol/ethyl acetate	[74]
	Farnesol acetate	Ethanol/ethyl acetate	[74]
	Ferulic acid-hexoside	Methanol	[76]
	Feruloylquinic acid	Methanol	[76]
	Friedelan-3-one	Ethanol/ethyl acetate	[74]
	Ferulic acid	Ethanol/ethyl acetate	[74]
	Gallic acid	Ethanol/ethyl acetate	[74]
	Gardenin	Methanol	[75]
	Germacratrienol isomer 1	Ethanol/ethyl acetate	[74]
	Germacratrienol isomer 2	Ethanol/ethyl acetate	[74]
	Germacratrienol isomer 3	Ethanol/ethyl acetate	[74]
	Hesperetin	Ethyl acetate	[73]
	Hydroxylated 4*β*-hydroxywithanolide E derivative	Ethanol/ethyl acetate	[74]
	Isoaromadendrene epoxide	Ethanol/ethyl acetate	[74]
	Isoferulic acid	Ethyl acetate	[73]
	Isorhamnetin	Ethanol/ethyl acetate	[74]
	Isovitexin	Methanol	[75]
	Kaempferol	Ethanol/ethyl acetate	[74]
	Kaempferol-3-O-rhamnosyl(1⟶6)glucoside	Methanol/water/formic acid	[76]
	Kaempferol-3-O-rhamnosyl(1⟶6)glucoside- 7-O-glucoside	Methanol/water/formic acid	[76]
	Kaempferol-hexoside	Ethanol/ethyl acetate	[74]
	Kaempferol-rutinoside	Ethanol/ethyl acetate	[74]
	Kamp3(2-p-manryl)glucose	Ethyl acetate	[73]
	Kamp3-7 di-rhamnoside	Ethyl acetate	[73]
	Khusiol	Ethanol/ethyl acetate	[74]
	Limonene	Ethanol/ethyl acetate	[74]
	Luteo 6 glucose 8 arabinose	Ethyl acetate	[73]
	Luteo 7 glucose	Ethyl acetate	[73]
	Maalialcohol	Ethanol/ethyl acetate	[74]
	Methyl-3,7-bis(acetyloxy)cholestan-26-oate	Ethanol/ethyl acetate	[74]
	Methylprednisolone succinate	Methanol	[75]
	Myricetin	Ethanol/ethyl acetate	[74]
	Naringin	Ethyl acetate	[73]
	Naringenin	Ethyl acetate	[73]
	O-Butanoyl-di-O-isobutanoylsucrose	Ethanol/ethyl acetate	[74]
	O-Decanoyl-O-isobutanoylsucrose	Ethanol/ethyl acetate	[74]
	O-Isobutanoyl-O-(2-methylbutanoyl)-O-octanoylsucrose	Ethanol/ethyl acetate	[74]
	O-Isobutanoyl-O-(2-methylbutanoyl)-O-pentenoylsucrose	Ethanol/ethyl acetate	[74]
	O-Isobutanoyl-O-(2-methylbutanoyl)sucrose	Ethanol/ethyl acetate	[74]
	O-Isobutanoyl-O-octenoylsucrose	Ethanol/ethyl acetate	[74]
	O-Isobutanoylsucrose	Ethanol/ethyl acetate	[74]
	*p*-Coumaric acid	Ethanol/ethyl acetate, ethyl acetate	[73, 74]
	Pheophytin a	Hexane/acetone/ethanol	[72]
	*p*-Hydroxy benzoic acid	Ethyl acetate	[73]
	Phytoene	Hexane/acetone/ethanol	[72]
	Phytol	Ethanol/ethyl acetate	[74]
	Protocatechuic acid	Ethanol/ethyl acetate, ethyl acetate	[73, 74]
	Pyrogallol	Ethyl acetate	[73]
	Quercetin	Methanol, ethanol/ethyl acetate, ethyl acetate	[74]
	Quercetin-3-O-glucoside	Methanol/water/formic acid	[76]
	Quercetin-3-O-rhamnosyl(1⟶6)glucoside-7-*O*-glucoside	Methanol/water/formic acid	[76]
	Quercetin-hexoside	Ethanol/ethyl acetate	[74]
	Quercetrin	Ethyl acetate	[73]
	Rhamncetin	Ethyl acetate	[73]
	Rosmarinic acid	Ethyl acetate	[73]
	Quercetin-3-O-rutinoside	Methanol/water/formic acid, ethanol/ethyl acetate	[74, 76]
	Salicylic acid	Ethyl acetate	[73]
	Sclareol	Ethanol/ethyl acetate	[74]
	Sclareol oxide	Ethanol/ethyl acetate	[74]
	Sesquichamene	Ethanol/ethyl acetate	[74]
	Sesquiterpeneol isomer	Ethanol/ethyl acetate	[74]
	Spironolactone	Methanol	[75]
	*trans*-Geranylgeraniol	Ethanol/ethyl acetate	[74]
	Tyrosol	Ethanol/ethyl acetate	[74]
	Vanillic acid	Ethanol/ethyl acetate	[74]
	Vanillin	Ethanol/ethyl acetate	[74]
	Vitexin	Methanol	[75]
	Withanolide D isomer	Ethanol/ethyl acetate	[74]
	Withanolide E isomer 1	Ethanol/ethyl acetate	[74]
	Withanolide E isomer 2	Ethanol/ethyl acetate	[74]
	Withanolide E isomer 3	Ethanol/ethyl acetate	[74]
	Xanthine	Methanol	[75]
	*α*-13,13-Dimethylpodocarp-7-en-3⍺-ol	Ethanol/ethyl acetate	[74]
	*⍺*-Coumaric acid	Ethyl acetate	[73]
	*⍺*-Elemol	Ethanol/ethyl acetate	[74]
	*α*-Tocopherol	Ethanol/ethyl acetate	[74]
	*α*-Tocopherol-*β*-D-mannoside	Ethanol/ethyl acetate	[74]
	*β*-Sitosterol	Ethanol/ethyl acetate	[74]
	*β*-Tocopherol	Ethanol/ethyl acetate	[74]
	*δ*-Cadinol	Ethanol/ethyl acetate	[74]
	*δ*-Terpineol	Ethanol/ethyl acetate	[74]
	*δ*-Tocopherol	Ethanol/ethyl acetate	[74]

*Fruits*	(-)-Caryophyllene oxide	—	[77]
	(5á)-Pregnane-3,20á-diol	Juice	[78]
	(9*Z*)-*β*-Carotene	Hexane/acetone/ethanol	[72]
	(all-*E*)-Antheraxanthin myristate-palmitate	Hexane/acetone/ethanol	[72]
	(all-*E*)-Lutein	Hexane/acetone/ethanol	[72]
	(all-*E*)-Lutein 3′-*O*-palmitate	Hexane/acetone/ethanol	[72]
	(all-*E*)-Lutein 3-O-myristate	Hexane/acetone/ethanol	[72]
	(all-*E*)-Lutein 3-*O*-palmitate-3′-*O*-myristate	Hexane/acetone/ethanol	[72]
	(all-*E*)-Lutein dimyristate	Hexane/acetone/ethanol	[72]
	(all-*E*)-Lutein dipalmitate	Hexane/acetone/ethanol	[72]
	(all-*E*)-Neoxanthin	Hexane/acetone/ethanol	[72]
	(all-*E*)-Neoxanthin dipalmitate	Hexane/acetone/ethanol	[72]
	(all-*E*)-Neoxanthin myristate	Hexane/acetone/ethanol	[72]
	(all-*E*)-Neoxanthin palmitate	Hexane/acetone/ethanol	[72]
	(all-*E*)-Taraxanthin	Hexane/acetone/ethanol	[72]
	(all-*E*)-Taraxanthin ester	Hexane/acetone/ethanol	[72]
	(all-*E*)-Violaxanthin	Hexane/acetone/ethanol	[72]
	(all-*E*)-Violaxanthin dimyristate	Hexane/acetone/ethanol	[72]
	(all-*E*)-Violaxanthin dipalmitate	Hexane/acetone/ethanol	[72]
	(all-*E*)-Violaxanthin myristate-palmitate	Hexane/acetone/ethanol	[72]
	(all-*E*)-Zeaxanthin dimyristate	Hexane/acetone/ethanol	[72]
	(all-*E*)-Zeaxanthin dipalmitate	Hexane/acetone/ethanol	[72]
	(all-*E*)-Zeaxanthin myristate-palmitate	Hexane/acetone/ethanol	[72]
	(all-*E*)-Zeinoxanthin	Hexane/acetone/ethanol	[72]
	(all-*E*)-*α*-Carotene	Hexane/acetone/ethanol	[72]
	(all-*E*)-*α*-Cryptoxanthin	Hexane/acetone/ethanol	[72]
	(all-*E*)-*α*-Cryptoxanthin myristate	Hexane/acetone/ethanol	[72]
	(all-*E*)-*α*-Cryptoxanthin palmitate		

	Palmitate	Hexane/acetone/ethanol	[72]
	(*E*)-2-Hexenol	—	[79]
	(*E*)-Non-2-enal	Dichloromethane	[80]
	(*E*)-*α*-Carotene	Hexane/acetone/ethanol	[72]
	(*E*2, *Z*6)-Nona-2,6-dienal	Dichloromethane	[80]
	(S)-4-Iodo-1,2-epoxybutane	—	[71]
	(*Z*)-Lutein 1	Hexane/acetone/ethanol	[72]
	(*Z*)-Lutein ester	Hexane/acetone/ethanol	[72]
	(*Z*)-Neoxanthin- or (*Z*)-violaxanthin ester	Hexane/acetone/ethanol	[72]
	(*Z*)-Stigmasta-5,24(28)-dien-3*β*-ol	Dichloromethane	[80]
	(*Z*)-Taraxanthin	Hexane/acetone/ethanol	[72]
	(*Z*)-*β*-Carotene	Hexane/acetone/ethanol	[72]
	(*Z*)-*γ*-Carotene	Hexane/acetone/ethanol	[72]
	∆5-Avenasterol	Crude oil	[81, 82]
	∆7-Avenasterol	Crude oil	[81, 82]
	1,1,1,5,7,7,7-Heptamethyl-3,3 bis(trimethylsiloxy) tetrasiloxane	—	[71]
	1,25-Dihydroxyvitamin D2	Juice	[71]
	1,2-Benzenedicarboxylic acid	—	[71]
	1,8-Menthadien-4-ol	—	[77]
	1-Phenyl-1,2-propanediol	—	[79]
	2,3-Diethyl-5-methyl pyrazine	Hexane and ethanol	[83]
	2,3-Dimethyl-1-butanol	—	[77]
	2-Acetyl-1-pyrroline	Dichloromethane	[80]
	2-Butanone	—	[77]
	2-Heptanol	—	[79]
	2-Heptanone	—	[77]
	2-Methylbutanal	—	[77]
	2-Methylbutanol	—	[77, 79]
	2-Methylbutanoic acid	—	[79]
	2-Methylbutyl acetate	—	[77]
	2-Methylpropanol	—	[79]
	2-Methylpropanoic acid	—	[79]
	2-Methylpropanal	Dichloromethane	[80]
	2-Methylpropenal	—	[77]
	2-Nonadecanol	—	[77]
	2-Norbornanone	—	[77]
	2-Pentanone	—	[77]
	2-Phenyl ethyl alcohol	Juice	[78]
	2-Phenylacetaldehyde	Dichloromethane	[80]
	2-Phenylethanol	Dichloromethane	[79, 80]
	2-Propanone	—	[77]
	2-Undecenal	Hexane and ethanol	[83]
	3,3-Dimethyl-hexane	—	[71]
	3,3-Dimethyl-octane	—	[71]
	3,4-Dimethylbenzoic acid	—	[71]
	3,5-Octadienone	Hexane and ethanol	[83]
	3,7-Dimethyl-1-octene	—	[77]
	3-Ethyl-4-heptanol	—	[77]
	3-Hydroxy-2-butanone	—	[79]
	3-Methyl-1-hexanol	—	[77]
	3-Methyl-1-penten-3-ol	—	[84]
	3-Methyl-3-vinyl-1-cyclopropene	—	[84]
	3-Methyl butyl butanoate	—	[77]
	3-Octenol	—	[77]
	3-Oxo-7,8-dihydro-*α*-ionol	Dichloromethane	[80]
	3-Phenyl propanol	—	[77]
	4-Hydroxy butyl acrylate	Hexane and ethanol	[83]
	4-Isopropyl-1-methyl-2-cyclohexen-1-ol	—	[77]
	4-Methyl-1-pentanol	—	[77]
	4-Nonanone	—	[77]
	4-Octanol	—	[77]
	4-Propyl guaiacol	Hexane and ethanol	[83]
	4-Terpineol	—	[77]
	4-Vinylguaiacol	—	[79]
	4-Vinylphenol	—	[79]
	4-Vinylsyringol	—	[79]
	4*β*-Hydroxywithanolide E	Hexane and ethanol	[83]
	5,6-Epoxy-*β*-carotene	—	[72]
	5,8-Epoxy-*α*-carotene	—	[72]
	6-Methyl-2-heptanone	—	[77]
	6-Methyl-5-heptene-2-one	—	[84]
	6-Methyl-hept-5-en-2-ol	—	[77]
	9-(Z)-Octadecenoic acid	—	[79]
	Acetaldehyde	—	[77]
	Acetic acid	—	[79]
	Allyl caproate	Hexane and ethanol	[83]
	Apigenin	Ethanol or water	[85]
	Apigenin 7 glucose	Ethyl acetate	[73]
	Benzaldehyde	—	[77, 84]
	Benzoic acid	Ethanol/ethyl acetate, ethanol, or water	[72, 85]
	Benzyl acetate	Hexane and ethanol	[83]
	Benzyl alcohol	—	[77, 79, 84]
	Betulin	Juice	[78]
	Butanal	—	[77]
	Butane-2,3-dione	—	[77]
	Butanoic acid	—	[77, 79]
	Butanol	—	[77, 79]
	Butanol-2-methyl	Hexane and ethanol	[83]
	Butyl 3-hydroxybutyrate	—	[84]
	Butyl acetate	Crude oil	[77, 84]
	Butyl butanoate	—	[77]
	Butyl decanoate	—	[77]
	Butyl dodecanoate	—	[77]
	Butyl octanoate	—	[77]
	Butyl-3-hydroxybutanoate	—	[77, 79]
	Caffeic acid	Methanol, ethanol/ethyl acetate	[78]
	Caffeine	Ethanol or water	[85]
	Campesterol	Dichloromethane	[86]
	Camphene	—	[77]
	Capric acid, methyl ester	—	[84]
	Carvacrol	—	[77]
	Caryophyllene oxide	—	[84]
	Catechin	Ethanol and isopropanol	[87]
	Catechol	Ethanol or water	[85]
	Cedr-8-en-9-alpha-ol acetate	Hexane and ethanol	[83]
	Cedrenol	Hexane and ethanol	[83]
	Chlorophyll a	Hexane/acetone/ethanol	[72]
	Chlorophyll b	Hexane/acetone/ethanol	[72]
	Chlorophyll b derivative 1	Hexane/acetone/ethanol	[72]
	Chlorophyll b derivative 2	Hexane/acetone/ethanol	[72]
	Cinnamic acid	-/ethanol or water	[72, 85]
	*cis*-3-Hexenol	—	[77]
	*cis*-Myrtanol	—	[77]
	*cis*-Piperitone oxide	—	[77]
	*cis-p*-Mentha-1(7),8-dien-2-ol	—	[77]
	*cis*-Verbenol	—	[77]
	Citronellyl acetate	Hexane and ethanol	[83]
	Cyclooctatetraene	—	[77]
	Cyclosativene	Hexane and ethanol	[83]
	Cymenene	—	[77]
	Decanal	—	[77]
	Decanoic acid	Juice, crude oil	[77, 79, 81, 82]
	Dehydrosabinene	—	[77]
	Diethyl ester	—	[71]
	Diethylene glycol	Methanol	[88]
	Dihomo-*γ*-linolenic acid	Crude oil	[81]
	Dihydroactinidiolide	—	[77]
	Dihydrocarveol	Hexane and ethanol	[83]
	Dimethylvinylcarbinol	—	[77]
	Docosane	—	[77]
	Docosanoic acid	—	[89]
	Dodecane	—	[84]
	Dodecanoic acid, methyl ester	—	[84]
	Eicosamethylcyclodecasiloxane	—	[71]
	Eicosanoic acid	Crude oil	[81, 82]
	Eicosenoic acid	Crude oil	[81, 82]
	Endo-borneol	—	[77]
	Epicatechin	Ethanol and isopropanol	[87]
	Erucic acid	Crude oil	[81, 82]
	Ergosterol	Crude oil	[81, 82]
	Ethanol	—	[77]
	Ethyl 2-methyl propanoate	Dichloromethane	[80]
	Ethyl acetate	—	[77]
	Ethyl benzoate	Juice	[78]
	Ethyl butanoate	Dichloromethane	[77, 80]
	Ethyl caprate	—	[84]
	Ethyl caproate	—	[84]
	Ethyl decanoate	—	[77]
	Ethyl dodecanoate	—	[77, 84]
	Ethyl hexanoate	Dichloromethane	[77, 80]
	Ethyl hexanol	—	[77]
	Ethyl hydroxyl hexanoate	—	[84]
	Ethyl octanoate	Dichloromethane, hexane, and ethanol	[77, 80, 83]
	Ethyl pentanoate	—	[77]
	Ethyl-2-butenoate	—	[77]
	Ethyl-3-hydroxybutanoate	—	[79]
	Ethyl-3-hydroxyhexanoate	—	[79]
	Ethyl-3-hydroxyoctanoate	—	[79]
	Ethyl-5-hydroxyoctanoate	—	[79]
	Eucalyptol	Hexane and ethanol	[77, 83]
	Farnesol	—	[77]
	Fenchol	—	[77]
	Ferulic acid	Methanol, ethanol/ethyl acetate	[78, 88]
	Furaneol	Dichloromethane	[80]
	Gallic acid	Ethanol and isopropanol, ethanol, or water	[85, 87]
	Geranaldehyde	—	[77]
	Geraniol	—	[77]
	Geranoic acid	—	[79]
	Geranyl acetone	—	[77]
	Guaiacol	—	[79]
	Heptan-2-ol	—	[77]
	Heptanal	—	[77]
	Heptanol	—	[77]
	Hexadecanoic acid	Crude oil, dichloromethane	[72, 76, 77]
	Hexadecanoic acid ester	Hexane and ethanol	[83]
	Hexanal	Crude oil, dichloromethane	[77, 80, 84]
	Hexanoic acid	—	[77, 79]
	Hexanol	—	[77, 79]
	Hexyl butanoate	—	[77]
	Hexyl ethanoate	—	[77]
	Hexyl octanoate	—	[77]
	Homofuraneol	Hexane and ethanol	[83]
	Hydrocinnamic alcohol	—	[77]
	Isoamyl octanoate	—	[77]
	Isobutyl acetate	—	[77]
	Isobutyl alcohol	—	[77]
	Isobutyl butanoate	—	[77]
	Isobutyl decanoate	—	[77]
	Isobutyl dodecanoate	—	[77]
	Isobutyl octanoate	—	[77]
	Isoeugenol	Hexane and ethanol	[83]
	Isophorone	—	[77]
	Isopropenyl ethyl ketone	—	[77]
	Isopulegol	—	[77]
	Kaempferol	Ethanol/ethyl acetate, ethanol, or water	[78, 85]
	Kaempferol 3-O-rutinoside	Juice	[78]
	Lanosterol	Crude oil	[81, 82]
	Limonene	Ethanol/ethyl acetate	[74, 77]
	Linalool	—	[77, 84]
	Linalool oxide	—	[77]
	Linoleic acid	Crude oil	[81, 82]
	Lucenin-2	Juice	[78]
	Lutein ester		[72]
	Methional	Dichloromethane	[80]
	Methyl acetate	—	[77]
	Methyl benzoate	—	[84]
	Methyl butanoate	Hexane and ethanol	[77, 83]
	Methyl butene	Hexane and ethanol	[83]
	Methyl decanoate	—	[77]
	Methyl heptenone	—	[77]
	Methyl hexanoate	—	[77]
	Methyl octanoate	—	[77]
	Methyl salicylate	—	[77, 79]
	Methyl *ß*-methylcrotonate	—	[84]
	Methyl-11-cyclopentylundecanoate	—	[77]
	Methyl-2-methoxyoct-2-enoate	—	[84]
	Methyl-3-hydroxybutanoate	—	[79]
	Myrcenol	—	[77]
	Neric acid	—	[77]
	Naringenin	Ethanol or water	[85]
	Nervonic acid	Crude oil	[81, 82]
	Neryl acetate	Hexane and ethanol	[83]
	Nonanal	—	[77]
	Nonanoic acid	—	[77]
	Nonanol	Hexane and ethanol	[83]
	Nopol	—	[77]
	O-Coumaric acid	Ethanol or water	[85]
	Oct-1-en-3-ol	Dichloromethane	[80]
	Octadecanoic acid	Crude oil	[81, 82]
	Octanal	-/Dichloromethane	[77, 80]
	Octanoic acid	—	[77, 79]
	Octanoic acid, 3-methylbutyl ester	—	[84]
	Octanol	—	[77]
	Oleic acid	Crude oil	[81, 82]
	Palmitoleic acid	Crude oil	[81, 82]
	*p*-Anisaldehyde	Hexane and ethanol	[83]
	*p*-Cymen-8-ol	—	[77]
	*p*-Cymene	—	[77]
	Pentyl alcohol	—	[84]
	Phenethyl alcohol	—	[77]
	Phenol	—	[79]
	Phenyl ethyl benzoate	Hexane and ethanol	[83]
	Phenylethyl acetate	—	[77]
	Pheophytin *b*	Hexane/acetone/ethanol	[72]
	*p*-Hydroxy benzoic acid	Ethanol or water	[85]
	Phytoene	Hexane/acetone/ethanol	[72]
	Phytofluene	Hexane/acetone/ethanol	[72]
	*p*-Menth-4(8)-ene-1,2-diol	—	[79]
	Propyl decanoate	—	[77]
	Propyl hexanoate	Hexane and ethanol	[83]
	Propyl octanoate	—	[77]
	Quercetin 3,4′,7-trimethyl ether	Juice	[78]
	Rosoxide	—	[77]
	Salicylic acid	Ethanol or water	[85]
	*sec*-Butyl butyrate	—	[77]
	Stigmasterol	Dichloromethane	[86]
	Syringic acid	Ethanol or water	[85]
	Terpinen-4-ol	—	[84]
	Terpinolene	—	[84]
	Tetradecanoic acid	Crude oil	[81, 82]
	Tetracosanoic acid	Crude oil	[81, 82]
	*trans*-3-Hexenol	—	[77]
	*trans*-Citral	—	[77]
	Trimethyl phenyl butenone	Hexane and ethanol	[83]
	Vanillic acid	Ethanol/Ethyl acetate, ethanol, or water	[74, 85]
	Vanillin	Ethanol/Ethyl acetate, ethanol, or water	[74, 85]
	Verbenene	Hexane and ethanol	[77, 83]
	Verbenone	—	[77]
	Vitamin B9 (folic acid)	Juice	[78]
	Vitamin E	Crude oil, dichloromethane	[81, 86]
	Vitamin K_1_	Crude oil	[81]
	*α*-Cubebene	Juice	[78]
	*α*-Linolenic acid	Crude oil	[76, 77]
	*α*-Pinene	Hexane and ethanol	[77, 83]
	*α*-Terpinene	—	[77]
	*α*-Terpineol	—	[77, 79, 84]
	*α*-Terpinolene	—	[77]
	*α*-Tocopherol	Crude oil, ethanol/ethyl acetate	[74, 81, 82]
	*β*-Bisabolol	Juice	[78]
	*β*-Carotene	Crude oil	[81, 82]
	*β*-Citronellol	—	[77]
	*β*-Cyclocitral	—	[77]
	*β*-Ionone	—	[77]
	*β*-Ionone-5,6-epoxide	—	[77]
	*β*-Linalool	Dichloromethane	[80]
	*β*-Myrcene	—	[77]
	*β*-Sitosterol	Crude oil	[81, 82]
	*β*-Tocopherol	Crude oil	[81, 82]
	*β*-*trans-*Ocimene	—	[77]
	*γ*-Butyl-*γ*-butyrolactone	—	[84]
	*γ*-Caprolactone	—	[84]
	*γ*-Ethylbutyrolactone	—	[77]
	*γ*-Linoleic acid	Crude oil	[81, 82]
	*γ*-Octalactone	Hexane and ethanol	[79, 83]
	*γ*-Terpinene	—	[77, 84]
	*γ*-Tocopherol	—	[82]
	*γ*-Undecalactone	—	[77]
	*δ*-Muurolene	Hexane and ethanol	[83]
	*δ*-Octalactone	—	[77, 79]

*Leaves*	(S)-4-Iodo-1,2-epoxybutane	—	[71]
	1,1,1,5,7,7,7-Heptamethyl-3,3	—	[71]
	1,2-Benzenedicarboxylic acid	—	[71]
	3,3-Dimethyl-hexane	—	[71]
	3,3-Dimethyl-octane	—	[71]
	Campesterol	Dichloromethane	[86]
	Diethyl ester	—	[71]
	Docosane	—	[77]
	Eicosamethylcyclodecasiloxane	—	[71]
	Ethyl isoallocholate	Dichloromethane	[86]
	Hexadecanoic acid	Dichloromethane	[86]
	Hexahydrofarnesyl acetone	Dichloromethane	[86]
	Linoleic acid	Dichloromethane	[86]
	Perulactone B	—	[90]
	Physalin B	—	[90]
	Physalin D	—	[90]
	Physalin F	—	[90]
	Phytol	Methanol, dichloromethane	[81, 86]
	Stigmasterol	Dichloromethane	[86]
	Vitamin E	Dichloromethane	[86]
	Withanolide E	—	[90]
	Withanolide F	—	[90]

*Peel*	(all-*E*)-Antheraxanthin myristate-palmitate	Hexane/acetone/ethanol	[72]
	(all-*E*)-Lutein	Hexane/acetone/ethanol	[72]
	(all-*E*)-Lutein 3′-*O*-palmitate	Hexane/acetone/ethanol	[72]
	(all-*E*)-Lutein 3-O-myristate	Hexane/acetone/ethanol	[72]
	(all-*E*)-Lutein 3-*O*-palmitate-3′-*O*-myristate	Hexane/acetone/ethanol	[72]
	(all-*E*)-Lutein dimyristate	Hexane/acetone/ethanol	[72]
	(all-*E*)-Lutein dipalmitate	Hexane/acetone/ethanol	[72]
	(all-*E*)-Neoxanthin	Hexane/acetone/ethanol	[72]
	(all-*E*)-Neoxanthin dipalmitate	Hexane/acetone/ethanol	[72]
	(all-*E*)-Neoxanthin myristate	Hexane/acetone/ethanol	[72]
	(all-*E*)-Neoxanthin palmitate	Hexane/acetone/ethanol	[72]
	(all-*E*)-Taraxanthin	Hexane/acetone/ethanol	[72]
	(all-*E*)-Taraxanthin ester	Hexane/acetone/ethanol	[72]
	(all-*E*)-Violaxanthin	Hexane/acetone/ethanol	[72]
	(all-*E*)-Violaxanthin dimyristate	Hexane/acetone/ethanol	[72]
	(all-*E*)-Violaxanthin dipalmitate	Hexane/acetone/ethanol	[72]
	(all-*E*)-Violaxanthin myristate-palmitate	Hexane/acetone/ethanol	[72]
	(all-*E*)-Zeaxanthin dimyristate	Hexane/acetone/ethanol	[72]
	(all-*E*)-Zeaxanthin dipalmitate	Hexane/acetone/ethanol	[72]
	(all-*E*)-Zeaxanthin myristate-palmitate	Hexane/acetone/ethanol	[72]
	(all-*E*)-Zeinoxanthin	Hexane/acetone/ethanol	[72]
	(all-*E*)-*α*-Carotene	Hexane/acetone/ethanol	[72]
	(all-*E*)-*α*-Cryptoxanthin	Hexane/acetone/ethanol	[72]
	(all-*E*)-*α*-Cryptoxanthin myristate	Hexane/acetone/ethanol	[72]
	(all-*E*)-*α*-Cryptoxanthin palmitate		

		Hexane/acetone/ethanol	[72]
	(*E*)-*α*-Carotene	Hexane/acetone/ethanol	[72]
	(*Z*)-Lutein 1	Hexane/acetone/ethanol	[72]
	(*Z*)-Lutein ester	Hexane/acetone/ethanol	[72]
	(*Z*)-Neoxanthin- or (*Z*)-violaxanthin ester	Hexane/acetone/ethanol	[72]
	(*Z*)-Taraxanthin	Hexane/acetone/ethanol	[72]
	(*Z*)-*β*-Carotene	Hexane/acetone/ethanol	[72]
	(*Z*)-*γ*-Carotene	Hexane/acetone/ethanol	[72]
	5,6-Epoxy-*β*-carotene	Hexane/acetone/ethanol	[72]
	5,8-Epoxy-*α*-carotene	Hexane/acetone/ethanol	[72]
	Lutein ester	Hexane/acetone/ethanol	[72]
	Phytoene	Hexane/acetone/ethanol	[72]
	Phytofluene	Hexane/acetone/ethanol	[72]

*Pulp*	(all-*E*)-Lutein	Hexane/acetone/ethanol	[72]
	(all-*E*)-Lutein 3-O-myristate	Hexane/acetone/ethanol	[72]
	(all-*E*)-Lutein 3-*O*-palmitate-3′-*O*-myristate	Hexane/acetone/ethanol	[72]
	(all-*E*)-Lutein dimyristate	Hexane/acetone/ethanol	[72]
	(all-*E*)-Lutein dipalmitate	Hexane/acetone/ethanol	[72]
	(all-*E*)-Neoxanthin	Hexane/acetone/ethanol	[72]
	(all-*E*)-Neoxanthin dipalmitate	Hexane/acetone/ethanol	[72]
	(all-*E*)-Neoxanthin myristate	Hexane/acetone/ethanol	[72]
	(all-*E*)-Taraxanthin	Hexane/acetone/ethanol	[72]
	(all-*E*)-Taraxanthin ester	Hexane/acetone/ethanol	[72]
	(all-*E*)-Violaxanthin	Hexane/acetone/ethanol	[72]
	(all-*E*)-Violaxanthin dimyristate	Hexane/acetone/ethanol	[72]
	(all-*E*)-Violaxanthin dipalmitate	Hexane/acetone/ethanol	[72]
	(all-*E*)-Violaxanthin myristate-palmitate	Hexane/acetone/ethanol	[72]
	(all-*E*)-Zeinoxanthin	Hexane/acetone/ethanol	[72]
	(all-*E*)-*α*-Carotene	Hexane/acetone/ethanol	[72]
	(all-*E*)-*α*-Cryptoxanthin	Hexane/acetone/ethanol	[72]
	(all-*E*)-*α*-Cryptoxanthin myristate	Hexane/acetone/ethanol	[72]
	(*E*)-*α*-Carotene	Hexane/acetone/ethanol	[72]
	(*Z*)-Lutein 1	Hexane/acetone/ethanol	[72]
	(*Z*)-Lutein ester	Hexane/acetone/ethanol	[72]
	(*Z*)-*β*-Carotene	Hexane/acetone/ethanol	[72]
	(*Z*)-*γ*-Carotene	Hexane/acetone/ethanol	[72]
	∆5-Avenasterol	Crude oil	[81, 82]
	∆7-Avenasterol	Crude oil	[81, 82]
	5,6-Epoxy-*β*-carotene	Hexane/acetone/ethanol	[72]
	5,8-Epoxy-*α*-carotene	Hexane/acetone/ethanol	[72]
	Campesterol	Crude oil	[81, 82]
	Decanoic acid	Crude oil	[81, 82]
	Eicosanoic acid	Crude oil	[81, 82]
	Eicosenoic acid	Crude oil	[81, 82]
	Erucic acid	Crude oil	[81, 82]
	Ergosterol	Crude oil	[81, 82]
	Hexadecanoic acid	Crude oil	[81, 82]
	Homo-*γ*-linolenic acid	—	[82]
	Lanosterol	Crude oil	[81, 82]
	Linoleic acid	Crude oil	[81, 82]
	Lutein ester	Hexane/acetone/ethanol	[72]
	Nervonic acid	Crude oil	[81, 82]
	Octadecanoic acid	Crude oil	[81, 82]
	Oleic acid	Crude oil	[81, 82]
	Palmitoleic acid	Crude oil	[81, 82]
	Phytoene	Crude oil	[81,82]
	Phytofluene	Crude oil	[81,82]
	Stigmasterol	Crude oil	[81, 82]
	Tetradecanoic acid	Crude oil	[81, 82]
	Tetracosanoic acid	Crude oil	[81, 82]
	*α*-Linolenic acid	Crude oil	[81, 82]
	*α*-Tocopherol	Crude oil	[81, 82]
	*β*-Carotene	Crude oil	[81, 82]
	*β*-Sitosterol	Crude oil	[81, 82]
	*β*-Tocopherol	Crude oil	[81, 82]
	*γ*-Linolenic acid	Crude oil	[81, 82]
	*γ*-Tocopherol	—	[82]
	*δ*-Tocopherol	Ethanol/ethyl acetate	[78]

*Roots*	(S)-4-Iodo-1,2-epoxybutane	—	[71]
	1,1,1,5,7,7,7-Heptamethyl-3,3 bis(trimethylsiloxy) tetrasiloxane	—	[71]
	1,2,3-Tri(t-butyl)cyclopropenylium tribromide	—	[71]
	1,2-Benzenedicarboxylic acid	—	[71]
	3,3-Dimethyl-hexane	—	[71]
	3,3-Dimethyl-octane	—	[71]
	3*α*-Tigloylnxytropane	Ethanol	[69]
	3*β*-Acetoxytropane	Ethanol	[69]
	Cuscohygrine	Ethanol	[69]
	Diethyl ester	—	[71]
	Dimethyl-flubendazole		
	Docosane	—	[71]
	Eicosamethylcyclodecasiloxane	—	[71]
	Hygrine	Ethanol	[69]
	N-Methylpyrrolidinylhygrine A	Ethanol	[69]
	N-Methylpyrrolidinylhygrine B	Ethanol	[69]
	Physoperuvine	Ethanol	[69]
	Tropine	Ethanol	[69]
	Dimethyl-flubendazole	—	[71]

*Seeds*	(S)-4-Iodo-1,2-epoxybutane	—	[71]
	1,1,1,5,7,7,7-Heptamethyl-3,3 bis(trimethylsiloxy)tetrasiloxane	—	[71]
	1,2-Benzenedicarboxylic acid	—	[71]
	3,3-Dimethyl-hexane	—	[71]
	3,3-Dimethyl-octane	—	[71]
	1,2,3-Tri(t-butyl) cyclopropenylium tribromide	Methanol	[88]
	Caffeic acid	Methanol	[88]
	Diethyl ester	—	[71]
	Diethylene glycol	Methanol	[88]
	Docosane	—	[77]
	Eicosamethyl cyclodecasiloxane	—	[71]
	Octadecanoic acid	Methanol	[88]

## Data Availability

All relevant data are presented in the manuscript. However, any required further information can be provided by the corresponding author upon request.
